# Cold-stress responses in the Antarctic basidiomycetous yeast *Mrakia blollopis*

**DOI:** 10.1098/rsos.160106

**Published:** 2016-07-06

**Authors:** Masaharu Tsuji

**Affiliations:** National Institute of Polar Research (NIPR), 10-3, Midori-cho, Tachikawa, Tokyo 190-8518, Japan

**Keywords:** cold stress, basidiomycetous yeast, metabolite response, capillary electrophoresis–time-of-flight mass spectrometry, *Mrakia blollopis*

## Abstract

Microbes growing at subzero temperatures encounter numerous growth constraints. However, fungi that inhabit cold environments can grow and decompose organic compounds under subzero temperatures. Thus, understanding the cold-adaptation strategies of fungi under extreme environments is critical for elucidating polar-region ecosystems. Here, I report that two strains of the Antarctic basidiomycetous yeast *Mrakia blollopis* exhibited distinct growth characteristics under subzero conditions: SK-4 grew efficiently, whereas TKG1-2 did not. I analysed the metabolite responses elicited by cold stress in these two *M. blollopis* strains by using capillary electrophoresis–time-of-flight mass spectrometry. *M. blollopis* SK-4, which grew well under subzero temperatures, accumulated high levels of TCA-cycle metabolites, lactic acid, aromatic amino acids and polyamines in response to cold shock. Polyamines are recognized to function in cell-growth and developmental processes, and aromatic amino acids are also known to improve cell growth at low temperatures. By contrast, in TKG1-2, which did not grow efficiently, cold stress strongly induced the metabolites of the TCA cycle, but other metabolites were not highly accumulated in the cell. Thus, these differences in metabolite responses could contribute to the distinct abilities of SK-4 and TKG1-2 cells to grow under subzero temperature conditions.

## 1. Introduction

Cold environments cover a large area on the Earth: approximately 85% of the biosphere is continuously exposed to temperatures below 5°C [1], and around 14% of this is contributed by the polar regions [2]. Antarctica, the southernmost landmass on the Earth, covers approximately 14 million km^2^, which makes it the fifth-largest continent in the world. Approximately, 98% of Antarctica is covered by ice and snow, and the temperatures in its coastal areas typically range from −35°C to 5°C; moreover, the temperatures on Antarctic plateaus are considerably more extreme: they range from around −70°C in winter to −25°C in summer [3].

Most culturable microorganisms are mesophiles and they occupy temperature niches that are not regarded as extreme. Conversely, psychrotrophic or psychrophilic microorganisms are especially adapted to low-temperature environments [4]. Microbes growing at low temperatures encounter several growth constraints: reduced enzyme reaction rates, transport-system efficiency and membrane fluidity, and increased stability of nucleic acid structures [5]. The ability of these microbes to thrive at low temperatures depends on numerous adaptations that are required to maintain the metabolic rates and sustained growth compatible with life in the cold [6].

Fungi that inhabit cold environments can grow and decompose organic compounds under subzero temperatures. Thus, cold-adapted fungi work as decomposers and play critical roles in the carbon cycle in polar-region ecosystems [7]. Consequently, understanding fungal cold-adaptation strategies under extreme environments is crucial for elucidating polar-region ecosystems.

Although the response to cold stress has been widely studied in bacteria and plants [8,9], little attention has been paid to fungal cold-adaptation strategies that are essential for survival at near subzero temperatures. Studies to date on fungal adaptation to low temperatures have examined whether or not the fungi growing under these conditions exhibits the physiological traits that are beneficial for survival in polar regions: accumulation of glycerol and trehalose in the cell, secretion of anti-freeze proteins and extracellular polysaccharides, and a high ratio of unsaturated fatty acids in the membrane [10]. In fungi, gene expression and metabolite accumulation in response to cold stress have typically been studied using the model microorganism *Saccharomyces cerevisiae*, which is considered a ‘conventional’ yeast [11,12]. Basidiomycetes were initially reported in early papers [13] and basidiomycetous yeasts have been widely reported to represent the dominant fungi in polar regions [14,15], and *S. cerevisiae* is categorized as an ascomycetous fungus. Basidiomycetous yeasts differ taxonomically from ascomycetous fungi.

The cold-adapted basidiomycetous yeasts *Mrakia* spp. and *Mrakiella* spp. have been found in the Arctic, Siberia, Alaska, Alps, Apennines, Patagonia and Antarctica [15–23]. Di Menna [13] reported that the genus *Mrakia* accounts for approximately 24% of the culturable fungi in Antarctic soil. Moreover, I previously reported that *Mrakia* spp. constitute approximately 35% of the culturable fungi isolated from the lake sediments and soils of East Antarctica [15]. My reports suggest that *Mrakia* spp. are the dominant culturable fungi near Syowa station, East Antarctica. Among this genus, *Mrakia blollopis* is the most frequently isolated species from the region and is well adapted to the environment.

In this study, I used capillary electrophoresis–time-of-flight mass spectrometry (CE-TOFMS) to analyse the metabolite responses to cold stress in two strains of the Antarctic basidiomycetous yeast *M. blollopis* under subzero temperatures. This is the first study to detect the metabolite changes in psychrophilic fungi under cold stress, and the results provide a clear overview of the metabolite responses elicited by cold shock in *M. blollopis*.

## 2. Material and methods

### 2.1. Yeast strains and media

In this study, two strains of *M. blollopis* were used, SK-4 and TKG1-2 [23], both of which were maintained on potato dextrose agar (PDA, Difco, Becton Dickinson Japan, Tokyo, Japan) at 4°C and inoculated on fresh PDA every two weeks.

### 2.2. Conditions for cultivation at −3°C and 10°C

*Mrakia blollopis* SK-4 and TKG1-2 were first cultivated in 20 ml of yeast extract--peptone--dextrose liquid medium (YPD, Difco, Becton Dickinson Japan) at 120 r.p.m. for 120 h at 10°C, following which the cells from the cultures were collected through centrifugation at 3500*g* for 5 min at 4°C. The cell pellets were resuspended in distilled water, and the washed cells were inoculated into 50 ml of modified YPD liquid medium (40 g l^−1^ glucose, 20 g l^−1^ peptone, 10 g l^−1^ yeast extract). For all cultivation conditions tested, the initial cell density in the YPD medium was adjusted to a 600 nm absorbance (OD_600_) of 2. Both strains were cultivated at 120 r.p.m. at −3°C and 10°C for 120 h, and 2–10 ml of each sample was collected once every 24 h and the glucose concentration in the supernatants was measured using high-performance liquid chromatography (HPLC). All experiments were conducted independently in four vials.

### 2.3. Determination of cell density and glucose concentration in the medium

Cell density was monitored by measuring the OD_600_ with a BioSpectrometer (Eppendorf, Hamburg, Germany), and the glucose concentration in the YPD liquid medium was determined by using an HPLC equipped with a refractive index detector (RI-2031Plus; JASCO, Tokyo, Japan) and an Aminex HPX-87H column (Bio-Rad Laboratories, Hercules, CA, USA). The HPLC equipment was operated at 65°C with a mobile phase of 8 mM H_2_SO_4_ at a flow rate of 0.6 ml min^−1^ [24].

### 2.4. Extraction of intercellular metabolites

Intracellular metabolites were extracted according to the method of Matsushika *et al.* [25] with minor modifications. To perform metabolome analysis on the yeast strains grown at −3°C and 10°C, SK-4 cells were collected once every 24 h from 0 h (control) up to 72 h, and TKG1-2 cells were harvested at 0 h (control) and 24 and 72 h; four samples were collected at each time point and their OD_600_ was measured. The culture volume used in the metabolome analysis was calculated using this formula:

$$\text{Required sampling volume (ml)}=\frac{20}{{\mathrm{OD}}_{600}}.$$

The collected required sampling volume was filtered by using a suction-filtering system equipped with an HTTP 0.4 µm pore filter (47 mm diameter Isopore Membrane Filter, Millipore, Billerica, MA, USA). Cells trapped on the filter were washed twice with 10 ml of MilliQ water and then soaked in 2 ml of methanol (MS analysis grade, Wako Pure Chemical Industries, Osaka, Japan) together with 5 µM internal standard on an encapsulated-type plastic dish. After 1 min, the dish was sonicated for 30 s (W-113 Sonicator, HONDA Electronics, Aichi, Japan) to suspend the cells completely, and then approximately 2 ml of the methanol solution including the cells and the internal standard was transferred to a 15 ml centrifugation tube (Corning Incorporated, Corning, NY, USA) and centrifuged at 2300*g* for 5 min at 4°C. The supernatant was transferred to an Amicon Ultrafree MC filter unit (Millipore) and centrifuged at 9100*g* for 120 min at 4°C. Lastly, the four filtrates obtained from the four vials were mixed in one tube, dried and then dissolved in 50 µl of MilliQ water.

### 2.5. Metabolite analysis and data processing

CE-TOFMS analysis was performed using an Agilent CE-TOFMS system (Agilent Technologies, Santa Clara, CA, USA). Cationic metabolites were separated in a fused silica capillary (50 µm i.d. × 80 cm total length) filled with a cation buffer solution (H3301-1001, Human Metabolome Technologies (HMT), Turuoka, Yamagata, Japan), and the sheath liquid (H3301-1020, HMT) was delivered. The sample solution was injected at 50 mbar for 10 s and a positive voltage of 27 kV was applied. Electrospray ionization--mass spectrometry (ESI-MS) was conducted in the positive mode and the capillary voltage was set at 4000 V. Exact mass data were acquired over a 50–1000 *m*/*z* range. Anionic metabolites were separated in a fused silica capillary filled with an anion buffer solution (H3302-1021, HMT), and the same sheath liquid mentioned above was delivered. The sample solution was injected at 50 mbar for 25 s and a positive voltage of 30 kV was applied. ESI-MS was conducted in the negative mode and the capillary voltage was set at 3500 V. Exact mass data were acquired over a 50–1000 *m*/*z* range. Peaks were extracted using the automatic integration software MasterHands v. 2.16.0.15 [26], and each metabolite was detected based on *m*/*z*, peak area, and migration time. Principal component analysis (PCA) and hierarchical clustering analysis were performed using SampleStat v. 3.14 (HMT) and PeakStat v. 3.18 (HMT).

## 3. Results

### 3.1. Glucose consumption and cell growth by two *Mrakia blollopis* strains

**Figure 1.**
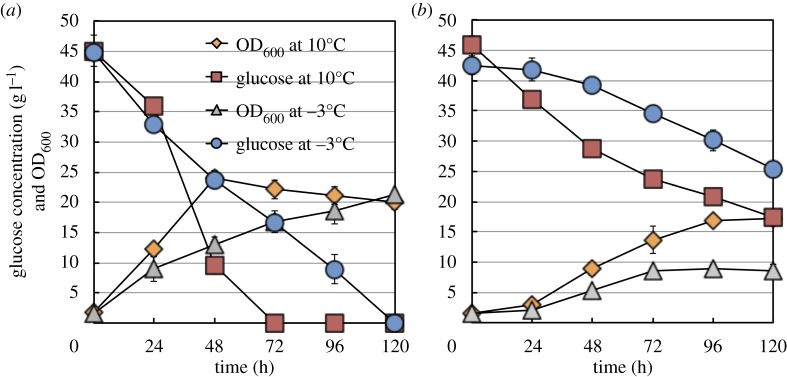
Cultivation time-dependent cell-growth and glucose-consumption profiles of *Mrakia blollopis* SK-4 and TKG1-2. Cell-growth and glucose-consumption profiles of *M. blollopis* strains cultivated at −3°C and 10°C: (*a*) SK-4 and (*b*) TKG1-2.

When SK-4 cells were grown at 10°C, glucose was completely consumed by 72 h and the OD_600_ peaked at 48 h and then gradually decreased up to 120 h, whereas at −3°C, glucose was consumed at a steady rate up to 120 h and the OD_600_ also increased up to 120 h. At 10°C and −3°C, the final OD_600_ values reached were almost the same (figure 1*a*). By contrast, when TKG1-2 cells were grown at 10°C, the glucose concentration decreased up to 120 h and approximately 14 g l^−1^ glucose remained in the medium, and the OD_600_ gradually increased until 96 h; moreover, at −3°C, glucose was consumed more slowly than at 10°C and approximately 25 g l^−1^ glucose remained in the medium, and the OD_600_ also increased more slowly than at 10°C (figure 1*b*).

### 3.2. Heatmap analysis and principal component analysis

Next, the effect of cold-shock stress on metabolite accumulation was examined in the two strains of *M. blollopis*. The metabolite response elicited by cold stress was determined by extracting and measuring metabolites in SK-4 and TKG1-2 cells at 4 and 3 time points, respectively, during the 120-h cultivation at −3°C and 10°C; sample collection, metabolite extraction and pooling, and CE-TOFMS analysis are detailed in §§2.4 and 2.5. In the CE-TOFMS analysis, 219 metabolites (115 cationic, 104 anionic) were detected. Moreover, 88 metabolites, which included amino acids, organic acids, sugar phosphates and nucleotides, were quantified using external standards and targeted metabolite analysis.

**Figure 2.**
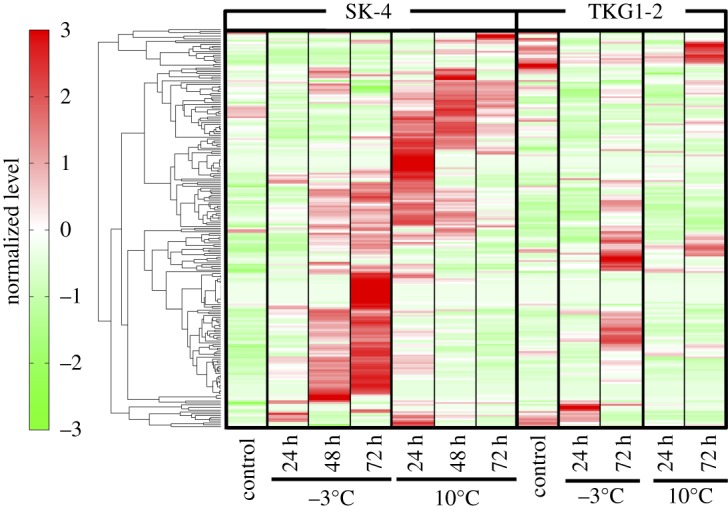
Heatmap analysis of metabolites in *M. blollopis* SK-4 and TKG1-2. Levels of all metabolites were standardized using mean 0 and variance 1. The normalized levels are shown according to colour on the left; deep-red and green represent the most increased and decreased metabolites.

After CE-TOFMS analysis, heatmap analysis and PCA were performed to determine whether or not cold stress induced changes in metabolite accumulation and responses. The results of the heatmap analysis showed that the metabolite levels in SK-4 were drastically altered by cold stress (figure 2), which affected, in particular, the central carbon metabolism pathway, the glycerol synthetic pathway, nucleic acids, aromatic amino acids and the polyamine synthetic pathway (electronic supplementary material, table S1). Therefore, the accumulation of these metabolites was investigated in detail.

**Figure 3.**
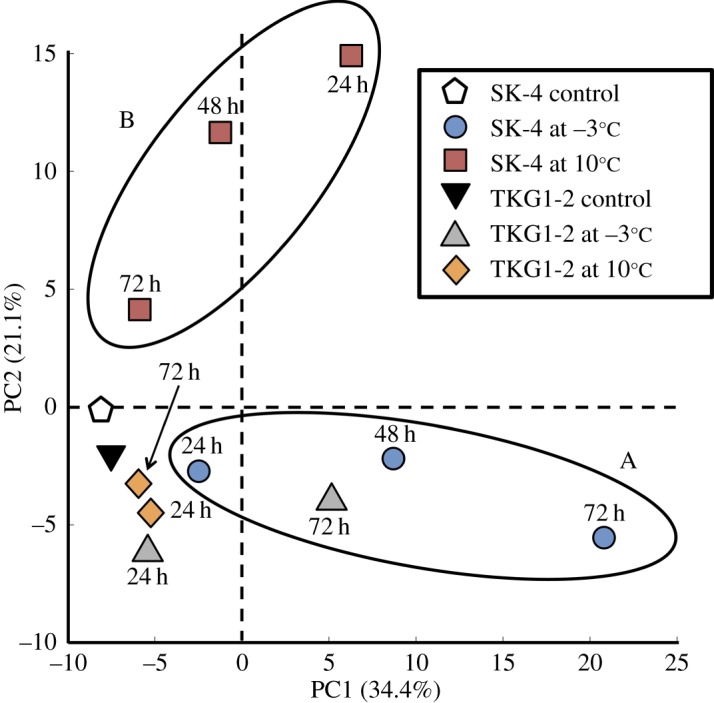
Principal component analysis of metabolite responses in *M. blollopis* SK-4 and TKG1-2. Results of PCA performed to examine changes in metabolite responses in SK-4 and TKG-1: the first and second components (PC1 and PC2) are shown.

The PCA results showed that the first two components accounted for approximately 55.5% of the total variance; whereas the first component (PC1) explained 34.4% of the variation, the second component (PC2) explained 21.1%. In the case of SK-4, the metabolite-response pattern for cold shock (A in figure 3) was clearly divided from the pattern at 10°C (B in figure 3); by contrast, the response pattern of TKG1-2 at 10°C was separated from the pattern at −3°C only at the 72-h cultivation time point.

### 3.3. Changes in central carbon pathway metabolites

**Figure 4.**
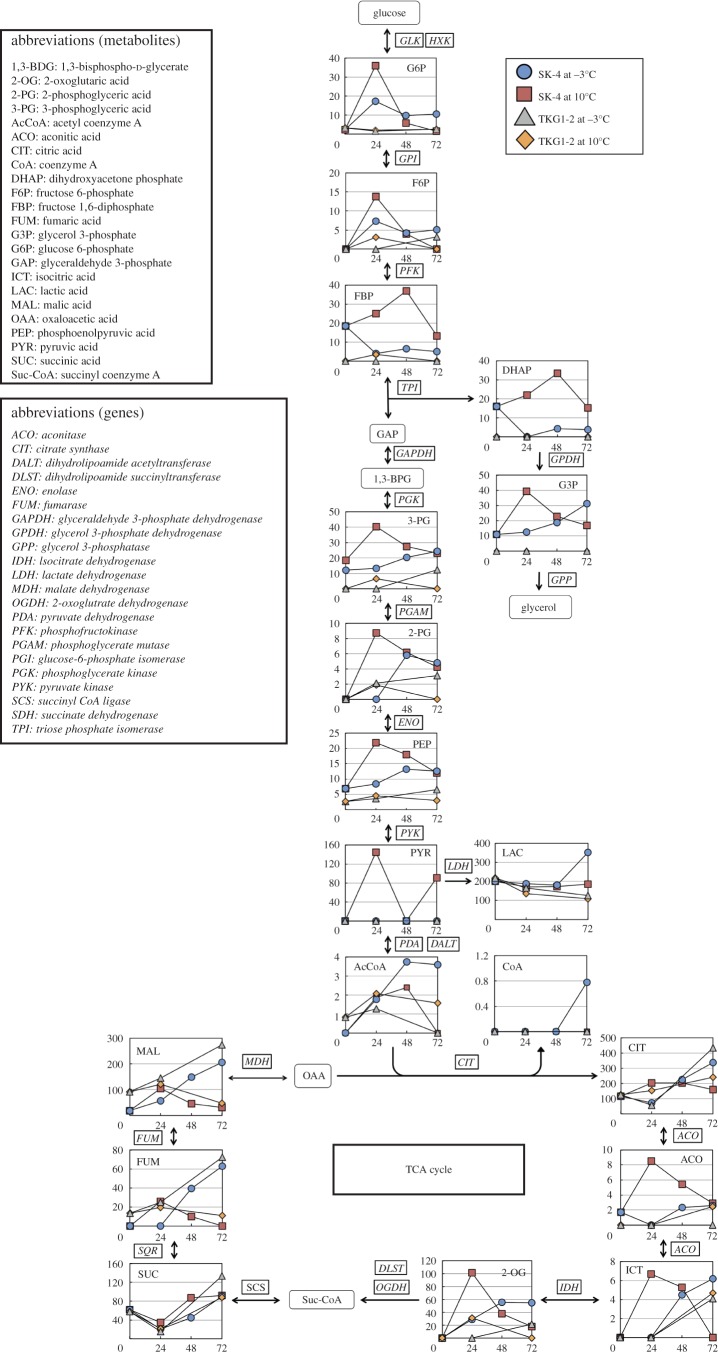
Comparison of metabolites in the central carbon pathway of *M. blollopis* SK-4 and TKG1-2. Arrows in the figure indicate the direction of enzyme reactions. Abbreviated names of metabolites are shown in the upper left part of the figure and of enzymes are shown in the middle left in italics. The *y*- and *x*-axes represent metabolite concentration (pmol/OD_600_ ml) and cultivation time (h).

The extent of metabolite changes in the central carbon metabolism pathway, including in glycolysis, the pentose phosphate pathway and the TCA cycle, is shown in figure 4. In the central carbon metabolism pathway, glyceraldehyde 3-phosphate, 1,3-bisphospho-d-glycerate (1,3-BPG), succinyl coenzyme A (Suc-CoA) and oxaloacetic acid were not detected. In SK-4 cells grown at 10°C, the following 11 of 19 metabolites in the central carbon pathway reached their peak concentration at 24–48 h and then decreased rapidly within the next 24 h: glucose 6-phosphate (G6P), fructose 6-phosphate (F6P), fructose 1,6-diphosphate (FBP), 3-phosphoglyceric acid (3-PG), 2-phosphoglyceric acid (2-PG), pyruvic acid, aconitic acid, isocitric acid, 2-oxoglutaric acid (2-OG), fumaric acid (FUM) and malic acid (MAL). By contrast, at −3°C, the FBP concentration was decreased after the first 24 h and then increased slightly; 3-PG gradually increased up to 72 h; and acetyl coenzyme A (AcCoA) and 2-PG markedly accumulated within 48 h. Moreover, lactic acid, AcCoA, CoA, FUM and MAL were accumulated to a greater extent at −3°C than at 10°C.

In the case of TKG1-2 cells cultivated at 10°C, the results obtained for the metabolites of the central carbon pathway were similar to those obtained for SK-4 cells; F6P, 3-PG, 2-OG, FUM and MAL were all detected, but most metabolites in TKG1-2 were scarcely accumulated as compared to the levels in SK-4 under both temperatures. However, citric acid, FUM and MAL in TKG1-2 cells showed similar accumulation as in SK-4 cells at −3°C.

### 3.4. Changes in metabolites in the glycerol and trehalose synthetic pathways

Glycerol is produced during glycolysis as follows: G6P is converted to FBP, which is converted to dihydroxyacetone phosphate (DHAP) and glycerol 3-phosphate (G3P) by triose phosphate isomerase and glyceraldehyde-3-phosphate dehydrogenase, and, finally, G3P is converted by glycerol-3-phosphatase (GPP) into glycerol, which either accumulates in the cell or is ejected into the extracellular medium (figure 4). In SK-4 cells cultivated at 10°C, DHAP was gradually accumulated up to 48 h, whereas G3P was rapidly synthesized within the first 24 h; the concentrations of these metabolites decreased over the next 24 h. By contrast, at −3°C, DHAP was decreased after the first 24 h and then slightly accumulated in the cells, much like FBP, whereas G3P was gradually increased up to 72 h. In TKG1-2 cells grown at 10°C and −3°C, the glycerol synthesis pathway metabolites DHAP and G3P could not be detected using CE-TOFMS analysis.

The trehalose biosynthesis pathway metabolites detected here were glucose 1-phosphate (G1P), uridine diphosphate glucose (UDP-glucose) and trehalose 6-phosphate (T6P); table 1 shows the relative concentrations of these metabolites. In SK-4 cells cultivated at −3°C, G1P was not detected at 0 h and it accumulated gradually over time, whereas at 10°C, G1P was accumulated after the first 24 h and then it decreased up to 72 h; by contrast, the G1P relative concentration was highest at 0 h in TKG1-2 cells. When the UDP-glucose peak area was defined as 1 at 0 h, its concentration relative to the corresponding control was found to be increased by more than 4.8-fold in SK-4 cells at 48 h and 2.6-fold in TKG1-2 cells at 72 h when the cells were cultivated at −3°C. By contrast, in SK-4 and TKG1-2 cells grown at 10°C, UDP-glucose peaked at 24 h at levels that were 4.4- and 1.3-fold higher than control, and then decreased to 1.4- and 1.1-fold, respectively. At −3°C, the T6P level was scarcely increased relative to control in either SK-4 or TKG1-2 cells, whereas at 10°C, T6P was decreased rapidly to 0.4- and 0.13-fold levels relative to control in SK-4 and TKG1-2 cells after the first 24 h, respectively, and then was increased to 1.7-fold in SK-4 cells at 48 h and 0.8-fold in TKG1-2 cells at 72 h.

**Table 1.** Relative concentrations of trehalose synthesis pathway metabolites produced by *M. blollopis* SK-4 and TKG1-2. Levels of each metabolite related to trehalose synthesis were calculated relative to control (0 h). N.D., not detectable.

	*M. blollopis* SK-4							*M. blollopis* TKG1-2				
	control	−3°C			10°C			control	−3°C		10°C	
	0 h	24 h	48 h	72 h	24 h	48 h	72 h	0 h	24 h	72 h	24 h	72 h
glucose 1-phosphate (G1P)	N.D.	>1	>1	>1	>1	>1	>1	1	0.6	0.8	0.5	0.8
UDP-glucose	1	3.6	4.8	3.9	4.4	1.7	1.4	1	1.4	2.6	1.3	1.1
trehalose 6-phosphate (T6P)	1	0.06	0.09	0.13	0.4	1.7	1.0	1	0.08	<1	0.13	0.8

### 3.5. Changes in aromatic amino acids

Table 2 shows the concentrations of aromatic amino acids in SK-4 and TKG1-2. Briefly, aromatic amino acids in SK-4 cells grown at −3°C increased continuously up to 72 h, whereas in TKG1-2 cells, they decreased for the first 24 h and then increased until 72 h. By contrast, at 10°C, the concentrations of these amino acids in SK-4 cells peaked at 24 h and then declined for the next 48 h, and in TKG1-2 cells, the concentrations decreased for the first 24 h and then increased until 72 h, as at −3°C, except in the case of histidine.

**Table 2.** Amounts of aromatic amino acids produced by *M. blollopis* SK-4 and TKG1-2 at various times at −3°C and 10°C.

	*M. blollopis* SK-4							*M. blollopis* TKG1-2				
	control	−3°C			10°C			control	−3°C		10°C	
	0 h	24 h	48 h	72 h	24 h	48 h	72 h	0 h	24 h	72 h	24 h	72 h
histidine (pmol/OD_600_ ml)	33	167	989	1,371	94	63	56	126	71	301	130	86
phenylalanine (pmol/OD_600_ ml)	30	51	111	199	117	84	47	26	27	77	11	9.5
tryptophan (pmol/OD_600_ ml)	2.3	5.7	7.9	9.2	5.9	4.4	3.3	3.6	3.2	4.3	1.4	1.8
tyrosine (pmol/OD_600 _ml)	6.2	21	45	52	24	13	8.6	9.9	9.3	20	4.6	4.1

In SK-4 cells cultivated at −3°C, the concentration of histidine increased drastically between 24 and 48 h, and finally was 41.5-fold higher than that at 0 h. Phenylalanine, tryptophan and tyrosine increased continuously and reached concentrations that were approximately 6.6-, 4.0- and 8.4-fold higher than those at 0 h, respectively. In TKG1-2 cells grown at −3°C, the concentration of histidine decreased to 0.56-fold and then increased to 2.4-fold relative to that at 0 h, whereas phenylalanine, tryptophan and tyrosine increased continuously and reached concentrations that were approximately 3.0-, 1.2- and 2.0-fold higher than those at 0 h, respectively. At 10°C, histidine concentrations in SK-4 and TKG1-2 cells increased for the first 24 h and finally decreased by 1.69-fold and 0.68-fold relative to the levels at 0 h, respectively. In SK-4 cells, phenylalanine, tryptophan and tyrosine concentrations peaked within 24 h and then dropped to levels that were approximately 1.6-, 1.4- and 1.4-fold higher than control; the concentrations of these amino acids in TKG1-2 also peaked at 24 h and then finally decreased by 0.37-, 0.50- and 0.41-fold relative to the control levels, respectively.

### 3.6. Changes in polyamine metabolites

**Figure 5.**
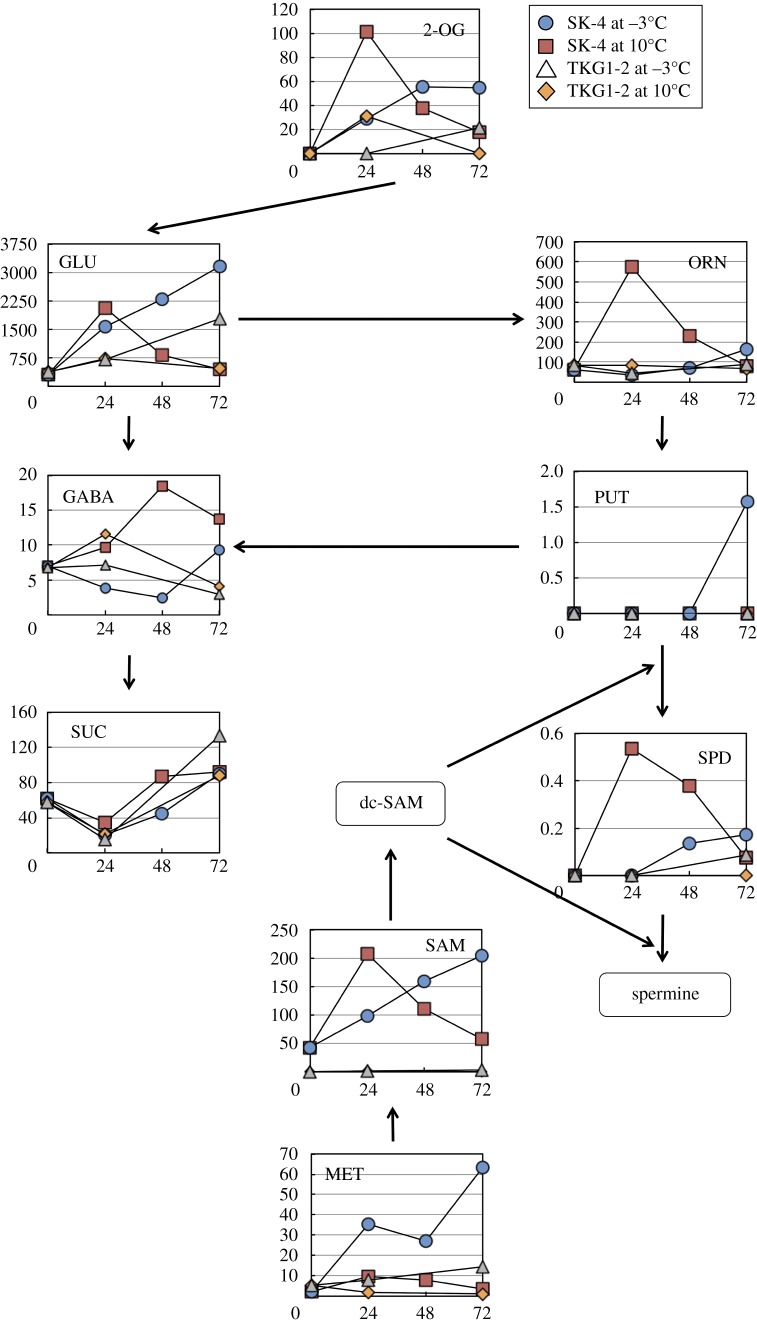
Profile of metabolites in the polyamine biosynthesis pathway of SK-4 and TLG1-2. Arrows in the figure indicate the direction of enzyme reactions. The *y*- and *x*-axes represent metabolite concentration (pmol/OD_600_ ml) and cultivation time (h), respectively. Abbreviations: 2-OG, 2-oxoglutaric acid; dc-SAM, decarboxylated *S*-adenosylmethionine; GABA, 4-aminobutanoic acid; GLU, glutamic acid; MET, methionine; ORN, ornithine; PUT, putrescine; SAM, *S*-adenosylmethionine; SPD, spermidine; SUC, succinic acid.

The changes in metabolite concentrations in the polyamine synthesis pathway are shown in figure 5. In SK-4 cells grown at −3°C, glutamic acid (GLU), methionine (MET) and *S*-adenosylmethionine (SAM) levels increased continuously and finally were 10.6-, 28.6- and 4.7-fold higher than the levels at 0 h. Furthermore, the highest concentrations of other metabolites in this pathway were recorded at 72 h. At 10°C, GLU, ornithine (ORN) and spermidine (SPD) concentrations peaked at 24 h, and ORN and SPD reached concentrations that were 3.5- and 3.1-fold higher than the corresponding maximal concentrations at −3°C. Moreover, 4-aminobutanoic acid (γ-aminobutyric acid, GABA) peaked at 48 h and its concentration was 2.0-fold higher than its highest concentration at −3°C. In TKG1-2 cells, the concentrations of polyamine metabolites at −3°C were almost the same as those at 10°C, but the concentration of GLU was 2.4-fold higher than its maximal concentration at 10°C.

### 3.7. Changes in energy charge

Adenylate energy charge (AEC) and guanylate energy charge (GEC) in SK-4 and TKG1-2 cells were calculated using this formula:

$$\mathrm{ACE}(\mathrm{GEC})=\frac{\left\{[\mathrm{ATP}(\mathrm{DTP})]+[\mathrm{ADP}(\mathrm{DDP})]\times 0.5\right\}}{\left\{\left[\mathrm{ATP}(\mathrm{DTP})]+[\mathrm{ADP}(\mathrm{DDP})\right]\}+[\mathrm{AMP}(\mathrm{DMP})\right]}.$$

Table 3 shows the amounts of adenine and guanine nucleotides and the ACE and GCE in SK-4 and TKG1-2. At 10°C, SK-4 ACE was 0.91 at 0 h and it remained above 0.84 at all cultivation times, whereas TKG1-2 ACE was 0.68 at first and then decreased to 0.56 at 72 h. At −3°C, SK-4 ACE decreased continuously and finally reached 0.72 at 72 h, whereas TKG1-2 ACE was maintained at the same level up to 72 h. Conversely, at 10°C, SK-4 GCE was maintained above 0.83 for up to 72 h, but TKG1-2 GCE decreased rapidly, to 0.64 within 24 h, and then remained almost at this level for the remainder of the cultivation period. At −3°C, SK-4 GCE was initially at the control level, but then decreased continuously to 0.74, whereas TKG1-2 GCE decreased to 0.68 after the first 24 h (as at 10°C) and then recovered to 0.74, which was the same charge level as in SK-4 at 72 h (table 3).

**Table 3.** Amounts of adenine and guanine nucleotides in *M. blollopis* SK-4 and TKG1-2 at −3°C and 10°C. Adenylate energy charge (AEC) and guanylate energy charge (GEC) were calculated according to this formula: ACE (GEC) = {[ATP (DTP)] + [ADP (DDP)]×0.5}/{[ATP (DTP)] + [ADP (DDP)]}+[AMP (DMP)]}. N.D., not detectable.

	*M. blollopis* SK-4							*M. blollopis* TKG1-2				
	control	−3°C			10°C			control	−3°C		10°C	
	0 h	24 h	48 h	72 h	24 h	48 h	72 h	0 h	24 h	72 h	24 h	72 h
ATP (pmol/OD_600_ ml)	40	53	83	84	96	98	65	38	28	54	32	33
ADP (pmol/OD_600_ ml)	4.8	12	33	42	25	14	15	24	18	35	24	31
AMP (pmol/OD_600_ ml)	1.6	3.1	12	19	7.5	6.2	6.1	12	7.8	14	14	23
GTP (pmol/OD_600_ ml)	7.7	9.2	18	16	24	22	8.2	6.0	3.7	11	5.5	6.9
GDP (pmol/OD_600_ ml)	2.5	2.8	7.0	8.0	6.7	5.6	4.1	3.0	2.4	5.3	3.4	3.9
GMP (pmol/OD_600_ ml)	N.D.	N.D.	1.4	2.9	1.2	1.6	N.D.	N.D.	1.1	2.1	2.3	3.4
ACE	0.91	0.86	0.78	0.72	0.84	0.89	0.84	0.68	0.69	0.69	0.63	0.56
GCE	0.88	0.88	0.81	0.74	0.86	0.85	0.83	0.83	0.68	0.74	0.64	0.62

## 4. Discussion

*Mrakia blollopis* strains SK-4 and TKG1-2 were isolated from, respectively, the lake sediment and soil collected near Syowa station in East Antarctica. These strains show greater than 99.5% similarity in the ITS (internal transcribed spacer) region and D1/D2 domain of 26S rDNA with the type strain of *M. blollopis*, CBS 8921 [23]. However, I previously reported that both strains exhibit distinct glucose-consumption characteristics and cell-growth abilities under low-temperature conditions [23,27]. Here, SK-4 and TKG1-2 were monitored for these two abilities under subzero conditions and then examined for their cellular metabolite responses.

SK-4 cells completely consumed the supplied glucose within 120 h at −3°C, and the OD_600_ of the cultures at −3°C reached almost the same value as that at 10°C; by contrast, in the case of TKG1-2 cells grown at −3°C, more than 25 g l^−1^ glucose remained after 120 h and the OD_600_ was approximately half that at 10°C (figure 1*a*,*b*). These results indicate that whereas the strain SK-4 is well adapted to subzero conditions, TKG1-2 does not grow efficiently at subzero temperatures. Accordingly, the metabolite responses induced by cold stress in these two strains were studied using CE-TOFMS. To the best of my knowledge, only a few studies have investigated the metabolite responses elicited by cold shock in fungi. Specifically, changes in the metabolites of the central carbon pathway have been only reported by López-Malo *et al.* [28], although the investigators did not examine the alterations in metabolite accumulation in accordance with cultivation time under cold-stress conditions. Here, in SK-4 cells grown at −3°C, AcCoA was markedly accumulated in the cell. Moreover, FUM and MAL were substantially accumulated in both SK-4 and TKG1-2 cells in response to cold stress, a phenomenon that was also reported in the case of *S. cerevisiae* grown in the presence of fermentation inhibitors [29].

Glycerol and trehalose are recognized as cryoprotectants that prevent the freezing of microbial cells at near-freezing temperatures [30]. In the final stage of the glycerol synthetic pathway, G3P is converted by GPP into glycerol, which is accumulated in the cell or ejected out of the cell. Interestingly, when *S. cerevisiae* was cultivated at a low temperature, glycerol was highly induced [31], but G3P was not substantially accumulated in the cell [28]. In the two Antarctic basidiomycetous yeast strains studied here, SK-4 and TKG1-2, cold shock did not strongly induce DHAP and G3P, which are intermediates in glycerol synthesis. In SK-4 cells grown at −3°C, G3P accumulated gradually over time, but the glycerol level peaked at 48 h (electronic supplementary material, table S1). Furthermore, the glycerol peak area was very small (see raw data in the dataset of the electronic supplementary material), and in a previous study, the gene encoding GPP was not found in the SK-4 genome [32]. Therefore, I propose that G3P was mostly converted into other metabolites related to the fatty acid synthesis pathway. With regards to cold-induced T6P accumulation, the peak area did not change in *S. cerevisiae*, but in cryotolerant species *Saccharomyces bayanus* var. *uvarum*, T6P was strongly induced by low temperature [28]. By contrast, the relative concentration of T6P in SK-4 and TKG1-2 was drastically decreased here under the subzero temperature used. Whereas *Mrakia* spp. are known to accumulate trehalose at low levels in the cell at low temperatures, *Glaciozyma antartica*, another Antarctic basidiomycetous yeast, accumulated approximately 20% (w/w) trehalose in the cell [33]. The two *Mrakia* strains studied here are considered to convert UDP-glucose into compounds in the glycogen synthetic pathway. SK-4 markedly induced lactic acid at −3°C, whereas TKG1-2 accumulated lactic acid to approximately one-third the level in SK-4. When *S. cerevisiae* NBRC 0308, which is known as sake yeast, was frozen in the presence of lactic acid at −20°C for 10 days at pH 5.0, the viability of the yeast cells was decreased to approximately 40%; however, when it was frozen in the absence of lactic acid, cell viability was decreased considerably more, to around 15% [34]. Based on my results and those of Togashi & Fukuda [34], I propose that SK-4 produces lactic acid to improve cell viability under subzero temperature conditions.

Polyamines are generally recognized to function in cell-growth and developmental processes [35]. The strain SK-4 was well adapted to growth below the freezing point, and, interestingly, in SK-4 cells, MET and SAM were accumulated at high levels. SAM was also strongly induced in *S. cerevisiae* under nitrogen- and sulfur-starvation conditions [36], and SAM is known to be related to ergosterol synthesis and to fungal cell-membrane constituents [37]. Therefore, I hypothesize that in SK-4, SAM accumulation bestows resistance to cold stress through ergosterol synthesis.

Aromatic amino acids, which have been reported to accumulate under cold, heat, acidity and oxidative stresses [38], are among the most costly compounds for the cell to synthesize in terms of ATP requirement [39]. In *S. cerevisiae*, aromatic amino acids are highly accumulated under carbon starvation and in the presence of fermentation inhibitors. Moreover, increased biosynthesis of aromatic amino acids, especially tryptophan, reflects the resistance of microbial cells to transport impairment and an improvement of cell growth at low temperature [40]. Here, histidine, phenylalanine, tryptophan and tyrosine were all strongly induced by cold stress in SK-4 cells, but not TKG1-2 cells, cultivated at −3°C. In SK-4, the ACE value decreased and the ADP/ATP ratio increased drastically in accordance with cultivation time at −3°C, which was caused by the increase in ADP and AMP concentrations. These results suggest that ATP, which is generated in the central carbon metabolism pathway, was consumed in the production of aromatic amino acids, and that ADP was consequently accumulated in the cell. By contrast, this phenomenon was not observed in TKG1-2 cells, which did not grow efficiently under the subzero temperature.

Lastly, the genus *Mrakia* has been reported from low-temperature areas worldwide, but *Mrakia* spp. scarcely produce extracellular polysaccharides, accumulate glycerol and trehalose in the cell as cryoprotectants, and do not harbour genes encoding anti-freeze proteins [15,32]. However, *Mrakia* can grow below −10°C [41] and is a common genus in the continental Antarctic region [42]. My results indicate that the Antarctic basidiomycetous yeast *M. blollopis* isolated from Antarctica experiences strong cold-shock stress. Consequently, SK-4 cells, which grew efficiently under subzero conditions, accumulated high levels of TCA-cycle metabolites, lactic acid, aromatic amino acids and polyamines as an adaptation to resist cold shock. By contrast, TKG1-2 cells, which did not grow efficiently in subzero temperatures, strongly induced the metabolites of the TCA cycle, but did not accumulate other metabolites at high levels in response to cold-shock stress. These differences in metabolite responses could contribute to the distinct abilities of SK-4 and TKG1-2 cells to grow under subzero temperature conditions.

## Supplementary Material

Spp. table 1. Dataset of principal component analysis of Mrakia blollopis SK-4 and TKG1-2 Levels of all metabolites were standardised using mean 0 and variance 1. Deep-red and green represent the most increased and decreased metabolites.

## Data accessibility

The data used for this study have been deposited in Dryad as http://dx.doi.org/10.5061/dryad.h5v25.
